# Editorial: Physiological growth responses to light in controlled environment agriculture

**DOI:** 10.3389/fpls.2024.1529062

**Published:** 2024-12-06

**Authors:** Jason Lanoue, Xiuming Hao, Viktorija Vaštakaitė-Kairienė, Leo F.M. Marcelis

**Affiliations:** ^1^ Harrow Research and Development Centre, Agriculture and Agri-Food Canada (AAFC), Essex, ON, Canada; ^2^ Department of Plant Biology and Food Science, Vytautas Magnus University Agriculture Academy, Akademija, Lithuania; ^3^ Horticulture and Product Physiology, Wageningen University and Research, Wageningen, Netherlands

**Keywords:** light, greenhouse, indoor vertical farming, controlled environment agriculture (CEA), food production

Light is crucial for photosynthesis, carbon assimilation, biomass production, and plant yield. It also influences physiological and biochemical processes. The lighting environment is vital in controlled environment agriculture (CEA) to enhance growth and quality. Advancements in lighting technologies and the CEA industry have accelerated lighting research in plant science (Pattison et al., 2018). The Research Topic was oriented towards compiling research related to the impact of the light environment in CEA with respect to physiological, biochemical, and genetic responses of plants. The Research Topic garnered 10 original research papers authored by 38 researchers from around the globe, including experts from Australia, Canada, China, Croatia, Hungary, South Korea, the Netherlands, and the United States of America.

In high latitude countries, supplemental light is required during winter months to achieve the optimum daily light integral. We have reached point where fundamental plant science research can be translated to elicit specific morphological and physiological responses during production all while maximizing energy-use-efficiency and minimizing greenhouse gas emissions. This will help the CEA industry ensure sustainable year-round production of fresh fruits and vegetables to meet consumer demand.

A study by Lanoue et al. demonstrated that pepper plants can be grown under continuous lighting without the leaf injury associated with a 24-h photoperiod. In addition, it turned out that a dynamic lighting strategy was necessary for injury-free production under 24-h lighting. Applying blue and/or far-red light at night decreased phytochrome photostationary state, which increased internode length and caused a shade avoidance response; applying far-red at night elicited a significantly stronger response than applying it during the day. In this study, a treatment that provided white light during the day followed by both blue and far red during the night, is potentially the best continuous lighting for pepper production. This study shows the using a low intensity, long photoperiod ultimately culminates in reduced capital fixture and electricity costs.

In another study, Lanoue et al. found that increased carbohydrate content and ROS-scavenging capability, as well as decreased photosynthetic pigment content, may be an adverse response to continuous lighting treatment. However, pepper plants did not show any impact on yield, nor did they show a stress response using the current gold standard stress metric, dark-adapted chlorophyll fluorescence measuring quantum yield of photosystem II (F_v_/F_m_). Interestingly, light-adapted chlorophyll fluorescence measurements assessing quantum efficiency of photosystem II (ΦPSII) and electron transport rate (ETR) decreased under increasing nighttime light intensity. Due to the discrepancy between dark-adapted and light-adapted measurements, the researchers suggest that light-adapted measurements may be more suitable for identifying stress response in continuous light tolerant crops.

The 16-h and 24-h constant (no change in light intensity and spectrum) and two 24-h dynamic (involving changes in spectra and intensity at different timings of the day) lighting strategies were presented by Marie et al. In the study, the morphological response of tomato, photoperiodic injury–sensitive species, and mini-cucumber, a photoperiodic injury-tolerant species was investigated. Moreover, the hypothesis of photorespiration’s involvement in photoperiodic injury was tested. Different dynamic strategies induced different canopy responses, opening the potential to adjust canopy architecture through counterbalances in the peak spectrum (blue) and night spectrum (far-red). Both tomato and cucumber responded well to the dynamic 16-h “day”, 3-h “peak”, 8-h “night” spectra by avoiding the typical compact morphology induced by extended photoperiods. A central discovery was that this strategy had a significantly higher level of photorespiration than control. Unexpectedly, photorespiration was comparable between tomato and cucumber under the same treatments, except under constant 24-h treatment. According to preliminary data, a fully tolerant tomato genotype grown under constant treatment upregulated photorespiration like mini cucumber. These results suggest that photoperiodic injury tolerance involves a sustained higher level of photorespiration under extended photoperiods.

Darko et al. investigated how the two light intensities (300 and 500 μmol m^–2^ s^–1^) were applied in different spectral compositions - broad white LED spectrum with and without FR application and with blue LED supplement was compared to blue and red LED lightings in different (80/20 and 95/5) blue/red ratios - affect the growth, flowering, and yield of chili and the production of secondary metabolites. High light intensity increased harvest index (fruit yield vs. vegetative biomass production) and reduced flowering time. Phenolic content and radical scavenging activity was stimulated by blue light, while capsaicin accumulation was suppressed. The red color of the fruit, which is determined by content of carotenoids, was inversely related to the absolute amount of blue, green, and far-red light. These findings demonstrated that the accumulation of secondary metabolites may be altered by adjusting light fluence and spectral composition, but different spectral combinations are necessary to trigger the accumulation of various phytochemicals. It was concluded that a single spectral combination is insufficient for the optimal growth of chili and the accumulation of all metabolites, and an adjustable light environment can ensure such conditions in CEA.

The study by Naveed et al. aimed to evaluate and comprehend the impacts of diminished light intensity and quality on plant morphology and root growth. In addition, they strived to identify resistant sources from the population generated from two-drought tolerant commercial chickpea lines (Sonali as a female and PBA Slasher as a male parent). Low light conditions, created by covering one of the two benches inside two growth chambers with a mosquito net, reduced natural light availability by approximately 70%. The chickpea genotypes exhibited significant responses to these conditions, mostly altering their morphology by allocating more photosynthates to shoot growth at the expense of root growth. Shading resulted in taller plants with longer and more internodes, but with lower root, shoot, and total plant biomass, presumably as an adaptation strategy, akin to the shade avoidance syndrome concept. The findings help better understand the biomass partitioning patterns in crops exposed to low light conditions.

Another study on low-light-stress in plants was done by Cao et al. when the growth characteristics of cucumber seedlings under various LED (light emitting diode) light treatments were evaluated. Low-light-stress tolerant and sensitive cucumber lines were used as plant materials for gene expression analysis. Light intensity below 40 μmol m^-2^ s^-1^ can quickly induce low-light-stress response. A total of 11 photoreceptor genes were identified and evaluated. Among them, cryptochrome 1 had the highest expression level and was only induced in the low-light sensitive cucumber. Therefore, it was proposed that cryptochrome 1 plays a pivotal role in regulation low-light response in plants

Medical cannabis cultivation has expanded under controlled environments. Increasing inflorescence weight and specialized metabolite concentrations is crucial for product consistency. The interaction between spectrum and intensity on inflorescence weight and secondary metabolites is attracting attention. The findings by Holweg et al. showed that white light with dual red peaks at 640 and 660 nm increased inflorescence yield and light-use-efficiency (LUE) in medical cannabis plants, regardless of intensities. This was primarily due to increased total plant dry matter production and a more open plant architecture. No light spectrum or intensity effects on cannabinoid concentrations were observed. However, at higher intensity, white light with dual red peaks increased terpenoid concentrations. At low intensity, photosynthetic parameters like maximum photosynthetic rate and quantum yield increased, while spectrum had no effect at higher intensity. The addition of 640 nm and 660 nm shows potential for improving LUE and plant dry matter production.

Enhancing supplemental lighting increased photosynthesis and had a significant impact on the water usage dynamics in cannabis leaves and crops. The findings of Collado et al. highlight the potential of lighting management to enhance water-use-efficiency (WUE), with significant implications for both research and practical applications in agriculture. Light supplementation strongly enhanced photosynthesis and plant growth while increasing WUE. It was found that a linear growth response within the range of ~18 to 52 mol m^-2^ d^-1^. Additionally, it was found that 52 mol m^-2^ d^-1^ did not saturate the crop response to light, leaving further research to be done to identify the maximum daily light integral for cannabis cutting production.

In controlled environment agriculture, tailored light treatments using LEDs are crucial for enhancing crop quality and yield. In the study by Van Brenk et al., lettuce was grown under three different blue and red light ratios with and without far-red light. As the control treatments, white light with and without far-red light was used. Decreasing the red:blue ratio decreased fresh weight and carbohydrate concentration, whereas contents of pigments, phenolic compounds, and various minerals increased. In contrast, adding far-red light to different R:B ratios, and increase in plant fresh weight, dry weight, total soluble sugars, and starch was observed. Additionally, far-red light decreased concentrations of anthocyanins, phenolic compounds, and various minerals. Consequently, the distinct advantages of enhanced blue light proportion and additional far-red radiation can be integrated and utilized synergistically to cultivate crops of desired quality.

High planting densities result in increased light interception and harvestable yield per area, but at the sacrifice of product quality. The study of Karpe et al. aimed to maintain high light interception without negative impacts on tomato fruit quality. Dwarf tomato was grown at four densities: two constant densities (high and low) and two dynamic spacing treatments (maintaining 90% and 75% ground coverage by decreasing planting density in 3–4 steps). The study found that high ground coverage and light interception are crucial for maximizing yield per area and LUE. Plants grown at the constant high planting density utilized light most efficiently for fruit yield formation, but reduced fruit quality. Conversely, low planting density resulted in the lowest light interception and yield per cultivation area. Dynamic spacing, which involves growing plants at high planting density but spacing them apart to maintain constant ground coverages, resulted in the same fruit quality but doubled yield, thus mitigating density-induced trade-offs.

Controlled environment agriculture is a fast growing technology revolutionizing plant production. With its potential to enhance food security in harsh climates and provide consumers with fresh produce year-round, CEA offers significant promise. To unlock this potential, understanding the role of light is paramount. Lighting technologies are continuing to advance, expanding the possibilities for growers and researchers, enabling more diverse applications. However, in order to fully optimize growth, it is important to have continued research related to the interactions of light with other environmental parameters. Additionally, multi-disciplinary science studying the impact of light on pest and disease will allow for a comprehensive approach for producers. The manuscripts contained within the Research Topic serve as a foundation for these integrative advancements.
